# Case report: A golden tail of immunotherapy: significant tail effect in a chemotherapy-resistant advanced pulmonary sarcomatoid carcinoma patient treated by Sintilimab combined with Anlotinib

**DOI:** 10.3389/fimmu.2024.1452195

**Published:** 2024-11-06

**Authors:** Chenghao Fu, Haonan Du, Qiang Wang, Weiyou Zhu, Guangli Bian, Zhujuan Zhong, Yuheng Wang, Lei Cao

**Affiliations:** ^1^ Department of Oncology, The First Affiliated Hospital of Nanjing Medical University, Nanjing, Jiangsu, China; ^2^ Department of Thoracic Surgery, The First Affiliated Hospital of Nanjing Medical University, Nanjing, Jiangsu, China; ^3^ Department of Thoracic Surgery, Taizhou Fourth People’s Hospital, Taizhou, Jiangsu, China; ^4^ Department of Radiology, The Affiliated Suqian First People’s Hospital of Nanjing Medical University, Suqian, Jiangsu, China; ^5^ Department of Pathology, The Affiliated Suqian First People’s Hospital of Nanjing Medical University, Suqian, Jiangsu, China; ^6^ Department of Oncology, The Affiliated Suqian First People’s Hospital of Nanjing Medical University, Suqian, Jiangsu, China

**Keywords:** tail effect, immunotherapy, cancer therapy, PSC, ICI, Sintilimab

## Abstract

Tail effect is a unique phenomenon in immunotherapy characterized by the prolonged maintenance of therapeutic efficacy. It can be observable even after treatment cessation. Immunotherapy has gradually become a vital regimen for the treatment of advanced lung cancer patients, among which immune-combined therapies based on immune checkpoint inhibitors (ICIs) have been applied clinically and demonstrates considerable clinical efficacy. In this case report, the patient was pathologically diagnosed with pulmonary sarcomatoid carcinoma (PSC), a rare and highly aggressive subtype of non-small cell lung cancer (NSCLC) known for its poor prognosis due to high invasiveness and metastatic potential. After developing resistance to chemotherapy, the patient was treated with a combined regimen of sintilimab and anlotinib, leading to initial clinical improvement. Following just three cycles of this regimen, treatment was discontinued, and the patient was discharged. Remarkably, over the subsequent months, the patient exhibited a significant tail effect, evidenced by sustained therapeutic stability, continuous tumor regression, stable low levels of serum carcinoembryonic antigen (CEA), and further improvement in clinical symptoms. Tail effect is a golden tail of immunotherapy. This case illustrates that the tail effect of immunotherapy can offer substantial survival benefits for patients with unresectable advanced lung cancer who have failed chemotherapy.

## Introduction

Tail effect is a special phenomenon in clinical anti-tumor immunotherapy, referring to the long-term persistence of therapeutic efficacy, which continues to be observed even after treatment cessation. The underlying mechanism primarily involves the function of memory T cells (1–3), reflected at the molecular level by epigenetic regulation, long non-coding RNA (lncRNA), transcription factors, and cytokines (4). This phenomenon can provide considerable long-term clinical benefits to patients with advanced tumors, propelling immunotherapy to the forefront of advanced NSCLC treatment, and in some sense, altering the tumor progression pattern. In recent years, immunotherapy represented by ICIs has shown significant efficacy in the treatment of advanced NSCLC (5–7). Programmed death-1 (PD-1) inhibitory ICIs like sintilimab block the interaction between PD-1 and its ligand, programmed death-ligand 1 (PD-L1), thereby lifting the immune suppression imposed by tumor cells and activating T cell-mediated anti-tumor effects (8, 9). Combination regimens based on ICIs, including sintilimab combined with anlotinib, have been clinically applied (10–13). Anlotinib, a novel multi-target tyrosine kinase inhibitor, can inhibit angiogenesis and tumor cell proliferation, further enhancing the anti-tumor effects of ICIs (14, 15).

PSC is a rare and highly aggressive subtype of NSCLC, accounting for only 0.1%-0.4% of all lung malignancies (16, 17). Patients with PSC have a significantly higher risk of mortality compared to other types of NSCLC due to its high invasiveness and metastatic potential (16, 18). Chemotherapy is currently the primary treatment for advanced PSC, but its efficacy is often unsatisfactory due to the low sensitivity to chemotherapy and the rapid development of resistance (19–21). Other traditional treatment methods also have limited efficacy. Notably, PSC frequently exhibits high PD-L1 expression and high tumor mutation burden (TMB) (22), suggesting that it may benefit from ICIs. Several case reports have demonstrated significant efficacy of ICI-based combination therapies in advanced PSC patients (10, 23), potentially offering new treatment strategies for PSC.

This case report aims to illustrate the therapeutic process involving a combination of ICIs in the treatment of a patient with advanced, chemotherapy-resistant PSC, who exhibited a notable tail effect. This observation provides new perspectives on the comprehensive treatment strategies for patients with advanced NSCLC. While prior clinical trials have explored the combination of ICIs with anlotinib in PSC patients (23), this case is unique. It not only demonstrates the efficacy of combined ICIs therapy in an advanced chemotherapy-resistant PSC patient but also emphasizes the clinical benefit derived from the tail effect observed after the discontinuation of treatment. To date, this phenomenon has not been reported in PSC patients.

## Case presentation

**Figure 1:**
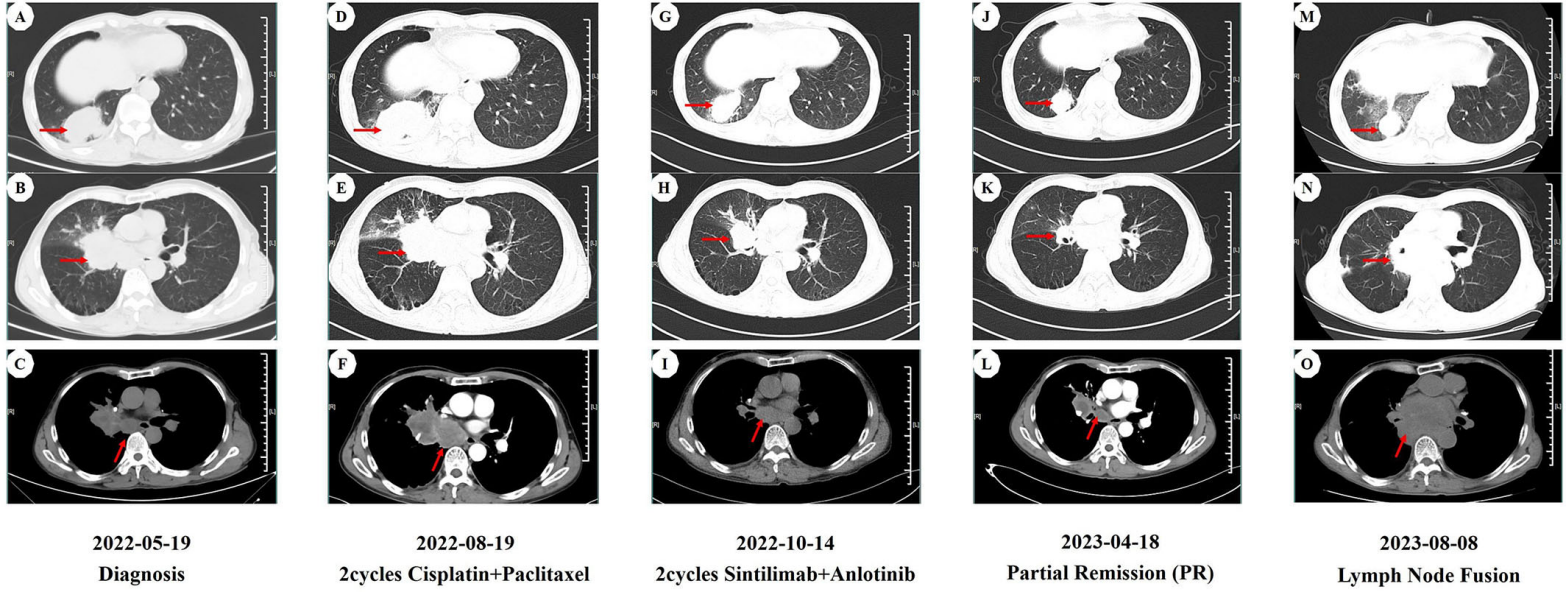
CT imaging assessment of lung lesions and mediastinal lymph nodes throughout the course of disease corresponding to their respective time points. **(A–C)** CT imaging of the lung window **(A, B)** and the mediastinal window **(C)** on May 19, 2022; **(D–F)** CT imaging of the lung window **(D, E)** and the mediastinal window **(F)** on August 19, 2022; **(G–I)** CT imaging of the lung window **(G,H)** and the mediastinal window **(I)** on October 14, 2022; **(J–L)** CT imaging of the lung window **(J, K)** and the mediastinal window **(L)** on April 18, 2023; **(M–O)** CT imaging of the lung window **(M, N)** and the mediastinal window **(O)** on August 8, 2023. **(A, D, G, J, M)** image manifests the lesion of the right lower lobe in the CT lung window. **(B, E, H, K, N)** image manifests the lesion of the right middle lobe in the CT lung window. **(C, F, I, L, O)** image manifests the mediastinal lymph nodes in the CT mediastinal window.

**Figure 2:**
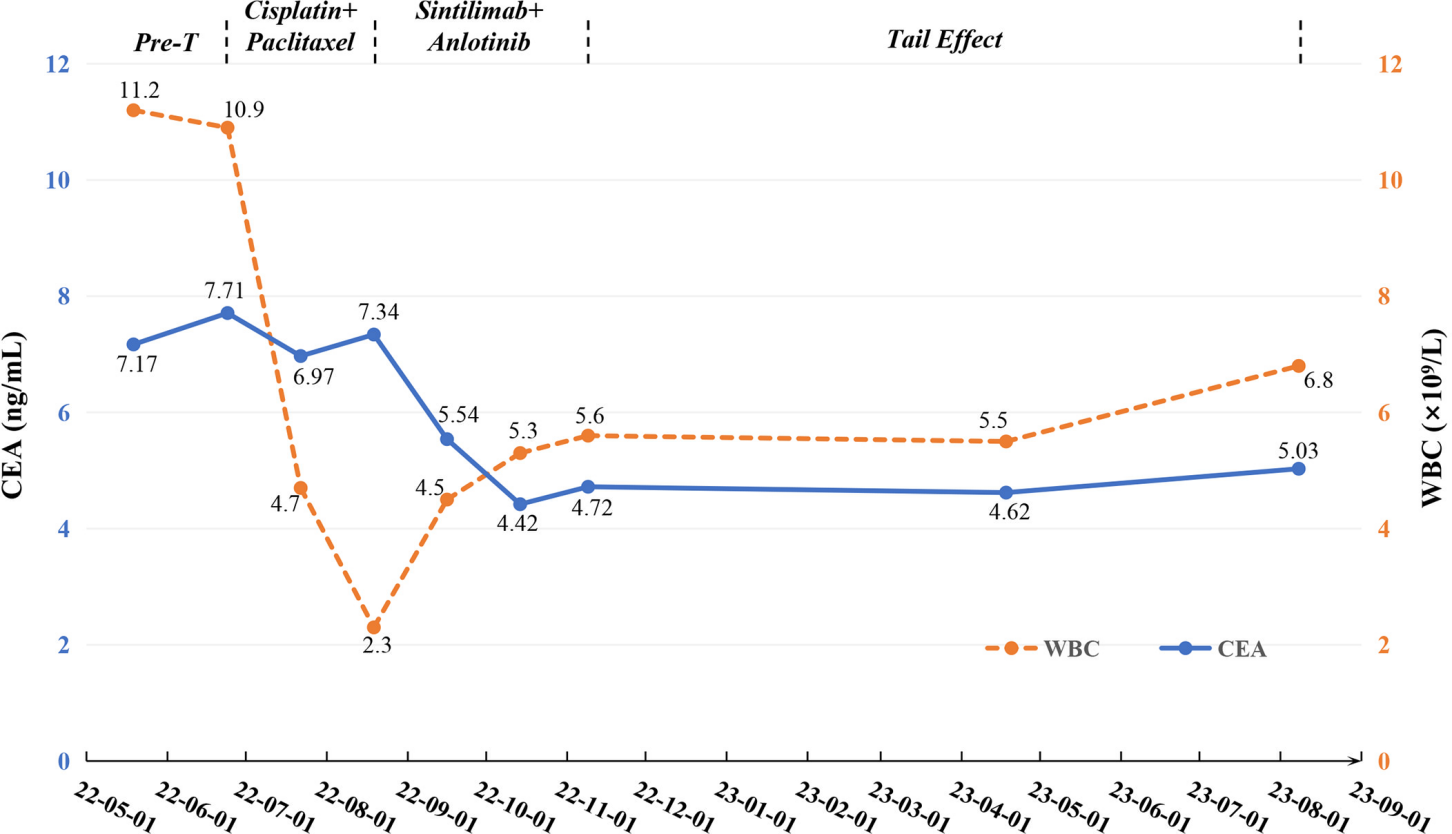
Changes of tumor markers and blood parameter during treatment. CEA, carcinoembryonic antigen; Pre-T, pre-treatment; WBC, white blood cell.

On May 19, 2022, a 59-year-old male patient presented with a one-month history of persistent dry cough and intermittent expectoration of white mucus. The patient had no family history of malignancy but had a history of smoking for over 30 years, with a smoking index of 600. He had recovered from pulmonary tuberculosis four years prior. The patient’s ECOG performance status was 1, and the Numeric Rating Scale (NRS) score was 3. A chest computed tomography (CT) scan revealed a soft tissue density shadow approximately 58*38 mm in size with bronchial obstruction in the right middle lobe (RML) and a high-density mass lesion approximately 71*53 mm in the right lower lobe (RLL) with adjacent bronchial obstruction, lobulation, and patchy shadows. Enlarged mediastinal lymph nodes measuring approximately 30*19 mm were observed. Localized pleural thickening was noted around the lesion (**Figures 1A–C**). Bone scans and enhanced cranial MR showed no metastases. CEA level was abnormally elevated at 7.17 ng/ml (reference range: 0-4.7 ng/ml). White blood cell (WBC) level was 11.2*10^9/L (**Figure 2**).

**Figure 3:**
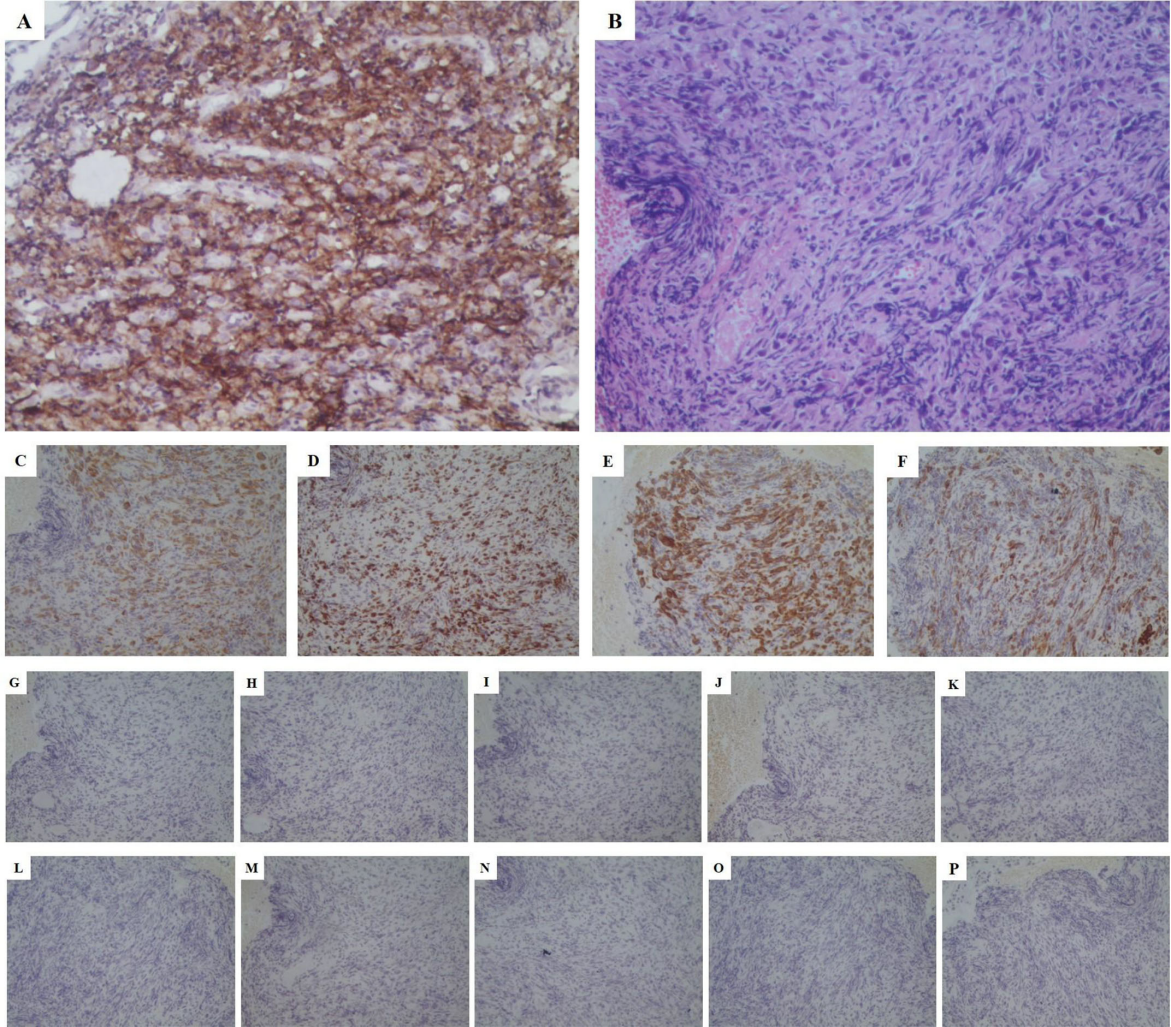
Pathological stained section (magnification:*200). **(A)** PD-L1 stained positive with a high expression (approximately 70%); **(B)** HE staining consistent with a poorly differentiated carcinoma, suggesting a sarcomatoid carcinoma phenotype; **(C)** CK-pan (+++); **(D)** Ki67 (50%+); **(E)** CK8/18 (+++); **(F)** CK7 (+++); **(G)** Napsin-A (-); **(H)** TTF-1 (-); **(I)** CgA (-); **(J)** SYN (-); **(K)** Cd56 (-); **(L)** Villin (-); **(M)** P63 (-); **(N)** P40 (-); **(O)** CK20 (-); **(P)** PSA (-). PD-L1, programmed death-ligand 1; HE, hematoxylin&eosin; CK, cytokeratin; TTF, thyroid transcription factor; SYN, synaptophysin; PSA, prostate specific antigen.

To further clarify the pathological nature, the patient underwent a bronchoscopic biopsy on May 21, 2022. Pathological results indicated malignancy in both lesions in the right middle and lower lobes, pending further immunohistochemical (IHC) analysis. IHC results showed negative staining for TTF-1, Napsin-A, SYN, CD56, CgA, Cd56, Villin, P63, P40,CK20 and PSA (**Figures 3G–P**)as well as positive staining for CK-pan (+++), Ki67 (50%+), CK8/18 (+++), and CK7 (+++) (**Figures 3C–F**). Combined with hematoxylin-eosin (HE) staining (**Figure 3B**), the case was consistent with a poorly differentiated carcinoma, suggesting a sarcomatoid carcinoma phenotype. Next-generation sequencing (NGS) revealed mutations in the KRAS and TP53 genes, but no mutations were detected in the EGFR, ALK, or MET exon 14 genes. Unfortunately, according to the CSCO guidelines for driver gene definition, this patient is unlikely to benefit from targeted therapies aimed at these specific mutations (24). After multidisciplinary consultation by department of oncology and pathology in our hospital and department of thoracic surgery in the first affiliated hospital of Nanjing Medical University, the patient was diagnosed with stage IVa poorly differentiated PSC involving the RML/RLL with pleural involvement (cT4N2M1 IVa stage).

Considering the unresectable state of the tumor, the patient began a first-line chemotherapy regimen of cisplatin combined with albumin-bound paclitaxel on June 24, 2022, receiving two cycles from June 24 to August 19, 2022. However, the patient experienced decreased appetite, nausea, vomiting, significant weight loss, and grade II leukopenia (WBC level: 2.3*10^9/L) (**Figure 2**), indicating poor tolerance to the chemotherapy’s toxic side effects. A follow-up chest CT on August 19, 2022, showed an increase in the size of the RML lesion to 61*37 mm, the RLL tumor to 73*56 mm, and mediastinal lymph nodes to 43 mm in diameter with partial fusion (**Figures 1D–F**). CEA levels further increased to 7.34 ng/ml (**Figure 2**) and the NRS score increased to 6. These signs indicated the appearance of chemotherapy resistance and progressive disease (PD).

In response to disease progression, we considered modifying the treatment regimen and evaluated the feasibility of immunotherapy. On August 20, 2022, with PD-L1 testing showing high expression (approximately 70%) (**Figure 3A**), we initially considered using sintilimab as ICIs medication and also took into account the favorable safety profile of anlotinib in combination therapy. Thus, the treatment regimen was finally adjusted to an immune-targeted combination of sintilimab and anlotinib. The patient received two cycles of treatment from August 22 to October 14, 2022, during which the adverse reactions to chemotherapy disappeared, and the patient reported symptom improvement. A follow-up chest CT after the second cycle on October 14, 2022, showed a reduction in the size of the lesions in the RML and RLL by 34.4% and 19.8%, respectively. The mediastinal lymph nodes also showed reduction and clearer contours (**Figures 1G–I**). CEA levels were controlled at 4.42 ng/ml, and WBC levels decreased to 5.3*10^9/L (**Figure 2**). The NRS score decreased to 4. The efficacy evaluation showed stable disease (SD), confirming the preliminary effect of the combination therapy. But due to personal willingness and financial reasons, the patient decided to discontinue treatment after completing the third cycle on November 9, 2022, and was discharged after signing informed consent.

Given the patient’s condition, we initially held a pessimistic outlook for follow-up results. However, surprisingly, during a follow-up visit on April 18, 2023, the patient exhibited a better mental state and further-improved clinical symptoms with a complete resolution of dry cough and chest pain, and an NRS score of 1. CT scans better confirmed the change: the RML lesion had shrunk to 27*21 mm, the RLL lesion to 51*31 mm, and the mediastinal lymph nodes to 21*19 mm (**Figures 1J–L**). The sizes were both better controlled than before: the RML lesion volume decreasing by 74% (compared with initial diagnosis) and 75% (compared with post-chemotherapy point), and the RLL lesion by 58% and 61%. CEA levels remained stable at 4.62 ng/ml, and WBC levels at 5.5*10^9/L (**Figure 2**). The efficacy evaluation showed partial response (PR). We considered this phenomenon the tail effect specific to immunotherapy. This is the first reported case of such a significant improvement: the patient’s condition continued to improve substantially after short-cycle medication and long-term discontinuation, indicating the sustained anti-tumor efficacy of immunotherapy, providing the patient with direct and significant survival benefits.

**Figure 4:**
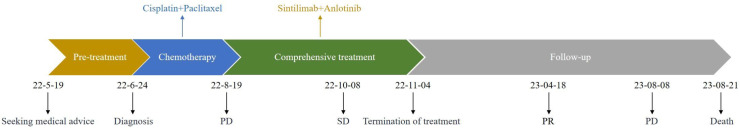
Patient treatment timeline.

Unfortunately, on August 8, 2023, a follow-up chest CT showed that the RML/RLL lesions remained well-controlled, and the NRS score remained at 1. CEA levels were 5.03 ng/ml (reference range: 0-4.7 ng/ml), and WBC levels were 6.8*10^9/L (**Figure 2**), maintaining good levels. However, the mediastinal lymph nodes showed abnormal enlargement and extensive irreversible fusion (**Figures 1M–O**). The patient, optimistic about his condition, refused further evaluation and passed away two weeks later at another hospital due to acute respiratory failure caused by a lung infection. The patient’s treatment timeline is shown in **Figure 4**.

## Discussion

Tail effect is a unique phenomenon associated with immunotherapy, referring to the prolonged maintenance of therapeutic efficacy which can be observable even after treatment cessation, providing long-term immune response and survival benefits for patients with advanced tumors. In this case, after the patient was diagnosed with PSC and exhibited chemotherapy resistance with disease progression; but initial clinical improvement was achieved following two cycles of immune-targeted combination therapy, with the efficacy evaluation being SD. Chemotherapy-induced adverse reactions disappeared, and pain was reduced. After completing three cycles of combination therapy, the patient discontinued treatment, but sustaining benefits continued to be observed for several months, with a significant tail effect: tumor size reduction, reversal of lymph node enlargement, substantial improvement in clinical symptoms, and a decrease in serum CEA levels. The efficacy evaluation was PR. This patient’s significant clinical improvement after short-cycle medication and long-term discontinuation represents the first typical report of this kind.

Most current studies suggest that tail effect is primarily related to the function of memory T cells (1–3). Tissue-resident memory T cells (TRM) can persist in the tumor microenvironment for a long time and initiate specific immune responses upon encountering tumor-specific antigens, producing anti-tumor effects (1, 3). Stem memory T cells (TSCM) and central memory T cells (TCM) ensure their long-term survival and maintain anti-tumor immune responses through self-renewal and multi-directional differentiation (1, 2). Their downstream molecular mechanisms include histone modifications, chromatin remodeling, and the role of long non-coding RNA(lncRNAs) (4). H3K4me1 marks in enhancer regions help maintain an open chromatin state, promoting gene transcription while H3K4me3 marks in promoter regions facilitate efficient transcription initiation (25, 26). These mechanisms concurrently enable rapid activation of memory T cells and immune responses. Chromatin remodeling makes the chromatin structure of specific genes more relaxed, increasing transcription efficiency. Certain lncRNAs, such as UMLILO, guide histone modification enzymes WDR5 and MLL5 to specific genes, enhancing their H3K4me3 marks and increasing their sensitivity during immune responses (4, 27). Additionally, transcription factors like STAT1 rapidly activate via the JAK-STAT pathway and mediate chromatin opening in promoter or enhancer regions, participating in the efficient activation of memory T cells (26, 28–30). Meanwhile, the establishment of memory domains ensures the persistence of immune memory and the high reactivity of TRM. Memory domains refer to gene regions that remain open after immune activation, allowing rapid transcription initiation upon recognition and binding by specific transcription factors. These domains remain open after the immune response ends, ensuring rapid immune responses to specific antigens later (4, 31). The synergetical effects of these mechanisms ensure the long-lasting and effective immune responses mediated by memory T cells, clinically manifested as tail effect.

The presence of tail effect highlights the potential value of immunotherapy for treating advanced tumors. Traditional chemoradiotherapy focuses on tumor-related cellular changes, often resulting in rapid elimination upon exposure; in contrast, immunotherapy can maintain a certain level of anti-tumor activity through the sustained presence of therapeutic effects, providing considerable long-term clinical benefits to patients with advanced tumors and altering tumor progression models to some extent. Therefore, immunotherapy offers new prospects for treating chemotherapy-resistant unresectable tumors. It relies on detecting specific biomarkers to select suitable candidates, with PD-1 and its ligand PD-L1 receiving particular attention. Currently, PD-L1 expression level testing has been most widely used. Numerous domestic and international immunotherapy studies have explored the guidance provided by PD-L1 expression levels on treatment regimens, with a series of results confirming the close correlation between PD-L1 expression levels and the efficacy of immunotherapy (32–34). PD-1 is expressed on T cells, while PD-L1 is often expressed on tumor cells. The binding of PD-L1 to PD-1 activates signaling pathways that inhibit T cell proliferation and reduce cytokine secretion, mediating tumor cell evasion of T cell immune responses (8, 9). Studies have confirmed that PSC patients exhibit high PD-L1 expression, approximately 40% higher than conventional NSCLC (35, 36). Additionally, PD-L1 expression is associated with aggressive pathological features such as N2 involvement, local, and distant metastases (37). Thus, blocking the PD-1/PD-L1 pathway can relieve T cell inhibition and achieve anti-tumor effects.

PSC is a pathological subtype of NSCLC with extremely poor prognosis, characterized by biphasic nature, epithelial and sarcomatoid components, and epithelial-mesenchymal transition (EMT) during growth (38), leading to decreased cellular adhesion and increased migratory capacity (39). This feature causes cancer cells to invade surrounding stroma from the primary tumor (40), exhibiting high invasiveness and metastatic potential. There is currently no individualized treatment guideline for PSC, with treatment strategies typically referring to NSCLC. Advanced PSC is often treated with platinum-based chemotherapy.

the patient initially received two cycles of chemotherapy but experienced disease progression and adverse reactions such as leukopenia, indicating chemotherapy resistance. Comparative studies indicate that chemotherapy has poor efficacy in PSC compared to conventional NSCLC and is prone to resistance (19–21). This suggests that the poor response to chemotherapy in PSC may be due to mechanisms such as continuous activation of EMT, leading to the spread of cancer cells to distant tissues and activation of tumor cells into cancer stem cells (CSCs) (41). CSCs, a rare subpopulation within tumors, contribute to intratumoral heterogeneity, a major cause of resistance (41). Wang’s study confirmed the enrichment of CSCs in cisplatin-resistant NSCLC cells (42). Additionally, KRAS and TP53 mutations have been identified as poor prognostic factors in PSC, increasing genomic instability and reducing response to chemotherapy, leading to poor treatment outcomes (10, 43). The German Lung Cancer Genomic Medicine National Network (nNGM) study (44) found that patients with co-existing KRAS and TP53 mutations are more likely to develop chemotherapy resistance, consistent with the patient’s case, suggesting the potential impact of genetic mutations on chemotherapy efficacy. Despite platinum-based doublet chemotherapy being the optimal first-line recommendation for advanced PSC patients, achieving satisfactory outcomes remains challenging. Therefore, the poor efficacy and adverse reactions to chemotherapy in advanced PSC patients highlight the urgent need for alternative treatment options.

Following poor response and adverse reactions to first-line chemotherapy, we rapidly conducted PD-L1 testing, obtaining high expression results, and selected the immune-targeted combination therapy of sintilimab and anlotinib after comprehensive evaluation of immunotherapy’s specificity and safety (11, 12, 45, 46). Sintilimab, a highly selective human monoclonal antibody, is approved as a first-line treatment for advanced NSCLC. It binds to the PD-1 receptor on T cells, blocking the interaction with PD-L1 on tumor cells, thereby disrupting the immune suppression reaction, promoting T lymphocyte activation, increasing CD4+/CD8+ and Th1/Th2, reducing Treg levels, reconstructing the tumor immune surveillance mechanism, preventing tumor cells from evading the immune system, and ultimately exerting anti-tumor effects (47). Vascular invasion is another significant characteristic of PSC, and anti-angiogenic targeted drugs like anlotinib may offer potential benefits for PSC patients. Anlotinib, an effective small molecule tyrosine kinase inhibitor, inhibits angiogenesis and the activation of VEGFR2, PDGFRb, and FGFR1, as well as their shared downstream ERK signaling pathway. It normalizes blood vessels, enhances immune cell infiltration, and regulates the composition of immune cells within tumor tissues, alleviating the immune-suppressive state in the tumor microenvironment (14). Preclinical studies show that sintilimab combined with anlotinib can reduce the activity of myeloid-derived suppressor cells and regulatory T cells, remodeling the tumor microenvironment, converting the immune-suppressive state to an immune-permissive mode, normalizing tumor blood vessels, promoting T cell infiltration into the tumor, enhancing immune function, and blocking immune suppressive signals through multiple pathways, increasing anti-tumor activity (15). The combination of these two drugs targets multiple mechanisms, achieving tumor immune killing and surveillance, promoting immune responses, and improving objective response rates (ORR) and disease control rates (DCR) (10, 13). A retrospective study demonstrated positive outcomes in NSCLC patients unresponsive to chemotherapy receiving sintilimab and anlotinib combination therapy (11). Domestic and international cases have shown excellent results with this combination therapy: Zhimin Zeng’s study confirmed that ICIs combined with anlotinib had better efficacy compared to chemotherapy or monotherapy (12). The phase 1b trial NCT03628521 reported an objective response rate of 72.7% and a median progression-free survival (PFS) of 15 months in 22 patients receiving sintilimab combined with anlotinib, with a significantly reduced incidence of treatment-related adverse events (TRAEs), indicating satisfactory and durable effects of the combination therapy (45). Peiliang Wang’s research specifically targeted chemotherapy-resistant advanced PSC patients, demonstrating that this combination therapy not only had low toxicity but also maximized disease improvement (11), consistent with the patient’s case: the patient showed significant improvement after receiving sintilimab and anlotinib, with disease control and substantial subjective symptom improvement.

Numerous clinical trials and case reports have indicated the existence of the tail effect in immunotherapy. A multicenter retrospective study found that patients achieving over six months of durable response from immunotherapy continued to benefit significantly after ICI discontinuation, showing long-term PFS (48). Phase III clinical trials KEYNOTE-024 and ASTRUM-005 compared PD-1 inhibitors with chemotherapy or placebo, showing similar survival curves: both PD-1 inhibitors produced significant differences from the control group around four cycles, with this difference increasing with extended treatment (49, 50), resulting in significantly better long-term survival outcomes for patients receiving immunotherapy. This indicates that immunotherapy’s long-term efficacy remains at a high level, providing substantial long-term clinical benefits to patients, aided by the tail effect. The CheckMate 227 trial compared immunotherapy and chemotherapy in advanced NSCLC patients, showing that 27% of patients in the immunotherapy group maintained response five years later, compared to only 4% in the chemotherapy group (51), further suggesting the tail effect. Yue Hu’s retrospective study analyzed treatment-free survival (TPS) in metastatic NSCLC (mNSCLC) patients after ICI discontinuation, finding that 35.5% of patients continued to benefit (52), with the tail effect during ICI-free periods providing nearly as much benefit as completing the treatment cycle, further confirming the widespread existence of tail effect and its high efficacy.

The significant tail effect observed in this case warrants exploration of the underlying mechanisms. The pronounced and persistent tail effect in this case may be related to PSC’s characteristics. PSC has high TMB and leukocyte fraction (LF), resulting in high neoantigen burden and T-cell inflamed tumor microenvironment (TME) (22), continuously stimulating T cells to produce specific immune responses, making PSC highly responsive to immunotherapy. Additionally, the pharmacokinetics and pharmacodynamics “disjunction phenomenon” of anti-PD-1 may contribute to the tail effect. A phase 1 study of anti-PD-1 (MDX-1106) found that although the drug’s half-life in serum is only 12-20 days, more than 70% of PD-1 molecules on circulating T cells remained occupied two months after infusion, regardless of the infusion dose (53). This suggests that even at very low serum concentrations, the therapeutic effect of anti-PD-1 can persist, ensuring sustained efficacy. The patient’s high PD-L1 expression also indirectly confirmed that blocking PD-1 with ICIs resulted in significant immune responses against tumor cells. Individual differences may also explain the patient’s favorable outcomes: the patient achieved significant clinical improvement after only two cycles of immune-targeted combination therapy, with an efficacy evaluation of PR after five months of discontinuation, which is rare among similar patients. Nana Huang reported a PSC patient undergoing continuous 10-cycle immune-targeted therapy, maintaining SD, but with less pronounced efficacy compared to this case (23).

It is undeniable that while this regimen has produced a significant tail effect, it also presents some inevitable drawbacks: despite the patient’s pulmonary lesion being evaluated as SD at the final follow-up, extensive fusion of the mediastinal lymph nodes was observed. This suggests that the combination of sintilimab and anlotinib may have limited efficacy in controlling lymph node lesions, potentially leading to lymph node recurrence or progression before the primary lesion. Additionally, the tail effect is difficult to quantify and monitor, and it may sometimes overlap with the effects of prior and subsequent treatments. The tail effect could also lead to patients and their families having an incomplete understanding of the disease status. Therefore, in clinical practice, we must balance risks and benefits, develop more detailed treatment plans, and enhance communication between doctors and patients about the treatment course of immune-targeted combination therapies.

The tail effect is often referred to as the “golden tail” of immunotherapy. As shown in this case, it has provided significant survival benefits for patients with chemotherapy-resistant or unresectable advanced lung cancer. In this case, the tail effect was maintained for several months following a short-term combination therapy regimen, and there are currently no reports of similar short-cycle treatment followed by long-term discontinuation. This further expands the available treatment options in clinical practice and holds important implications for future guidance. The diagnostic and therapeutic approach based on immune checkpoint inhibition may lead to breakthroughs in cancer treatment. However, based on this single case experience, more robust and comprehensive clinical trials or studies are needed to further validate the efficacy of these treatments.

## Data Availability

The original contributions presented in the study are included in the article/supplementary material, further inquiries can be directed to the corresponding authors.
