# Corrigendum: Case report: A golden tail of immunotherapy: significant tail effect in a chemotherapy-resistant advanced pulmonary sarcomatoid carcinoma patient treated by Sintilimab combined with Anlotinib

**DOI:** 10.3389/fimmu.2024.1527232

**Published:** 2024-11-26

**Authors:** Chenghao Fu, Haonan Du, Qiang Wang, Weiyou Zhu, Guangli Bian, Zhujuan Zhong, Yuheng Wang, Lei Cao

**Affiliations:** ^1^ Department of Oncology, The First Affiliated Hospital of Nanjing Medical University, Nanjing, Jiangsu, China; ^2^ Department of Thoracic Surgery, The First Affiliated Hospital of Nanjing Medical University, Nanjing, Jiangsu, China; ^3^ Department of Thoracic Surgery, Taizhou Fourth People’s Hospital, Taizhou, Jiangsu, China; ^4^ Department of Radiology, The Affiliated Suqian First People’s Hospital of Nanjing Medical University, Suqian, Jiangsu, China; ^5^ Department of Pathology, The Affiliated Suqian First People’s Hospital of Nanjing Medical University, Suqian, Jiangsu, China; ^6^ Department of Oncology, The Affiliated Suqian First People’s Hospital of Nanjing Medical University, Suqian, Jiangsu, China

**Keywords:** tail effect, immunotherapy, cancer therapy, PSC, ICI, Sintilimab

In the published article, there was an error in affiliations 4, 5, 6. Instead of “The First People’s Hospital of Suqian”, it should be “The Affiliated Suqian First People’s Hospital of Nanjing Medical University”.

The authors apologize for this error and state that this does not change the scientific conclusions of the article in any way. The original article has been updated.

